# Wildfire smoke exposure and cardiovascular disease—should statins be recommended to prevent cardiovascular events?

**DOI:** 10.3389/fcvm.2023.1259162

**Published:** 2023-09-14

**Authors:** Alpo Vuorio, Bruce Budowle, Frederick Raal, Petri T. Kovanen

**Affiliations:** ^1^ Mehiläinen, Airport Health Center, Vantaa, Finland; ^2^ Department of Forensic Medicine, University of Helsinki, Helsinki, Finland; ^3^ Faculty of Health Sciences, University of Witwatersrand, Johannesburg, South Africa; ^4^ Cardiovascular Research, Wihuri Research Institute, Helsinki, Finland

**Keywords:** wildfire smoke, endothelial dysfunction, acute myocardial infarction, cardiovascular disease, prevention, PM_2.5_

## Introduction

The risk of wildfires has been increasing which may well be associated with climate change (1, 2). The effects of wildfires can have serious consequences on human health. Currently, extremely destructive wildfires in Canada have generated substantial smoke within as well as beyond the borders of Canada (3). It has been estimated that toxic wildfire smoke from about 200 wildfires in Quebec alone, has affected over 100 million people in Canada and the USA (4). The severity of harm is such that residents in smoke-affected areas have been advised to use N95 masks when outdoors and, if possible, to remain indoors.

There are broad-based negative health effects caused by forest fires that will only worsen as their numbers and severity increase (5, 6). As an example, a study compared hospital visits during a wildfire in August 2015 and post-wildfire in September 2015 in the city of Calgary (7). In this study, physician visits increased by 19% among seniors, mainly related to ischemic heart disease (95% CI: 7%−33%). Wildfire smoke has also been associated with out-of-hospital cardiac arrest (8). Importantly, wildfire smoke has a significant negative impact on airway epithelial cell viability, as there is a disruption of the cellular survival processes governed by autophagy, i.e., of processes that are already dysregulated in chronic obstructive pulmonary obstructive disease (9). Moreover, even short-term exposures of pulmonary airways to PM_2.5_ particles cause systemic inflammation, oxidative stress, and changes in the balance of the autonomic nervous system, all of which may then jointly result in vascular thrombosis and ventricular arrhythmias (8, 10). The primary initiating pathways causing systemic oxidative stress and systemic inflammation include the migration of PM_2.5_ particles from the lungs to the circulation (11, 12), and as shown in a recent *in vivo* study, exposure to PM_2.5_ particles associates with features of vulnerable atherosclerotic plaques in coronary arteries (13).

It can be reasonably concluded that significant forest fires and smoke exposure are associated with a notable additional burden on healthcare services. Moreover, studies have revealed that several sectors of the population are particularly vulnerable to the effects of smoke from forest fires. As an example of social vulnerability, a study using satellite-collected data on wildfire smoke exposure at locations of exposed populations during 2011–2021 indicated that in almost 90% of the US population, large increases of exposure were especially high in communities representing ethnic minorities (14). In this article, we focus on another vulnerable group, i.e., people with cardiovascular disease.

Our intent was to perform a cursory assessment of public guidance that has been recommended for individuals with cardiovascular disease, hopefully, to prevent additional health-threatening events during exposure to wildfire smoke. The purpose is not to determine the effectiveness of current wildfire smoke health exposure guidelines (15), but simply to explore whether such guidelines contain evidence-based guidance for high-risk cardiovascular disease patients who are exposed to toxic smoke.

## Wildfire smoke exposure and cardiovascular events

In general, exposure to wildfire smoke has been shown to increase cardiovascular-related health issues such as acute myocardial infarction, cardiovascular mortality, cardiovascular emergency department visits and hospitalization, cardiac arrest, as well as heart failure (16–19). Especially vulnerable are those individuals who have pre-existing cardiovascular disease who, when exposed to external factors such as wildfire smoke-generated PM_2.5_ particles, can severely worsen their pre-existing endothelial dysfunction (20–22). Even acute exposure to PM_2.5_ particle pollution is associated with increased cardiovascular morbidity and mortality (18). This observation is due to inhaled particles entering the circulation and worsening systemic endothelial dysfunction which in turn causes adverse cardiovascular effects due to oxidative stress, inflammation, cytokine release, and hypercoagulability (23, 24). Additionally, diabetics are at increased risk from PM_2.5_ particle exposure because of pre-existing endothelial dysfunction as well as autonomic dysfunction due to diabetic neuropathy (20). Similarly, we proposed that patients with familial hypercholesterolemia, who have pre-existing endothelial dysfunction from lifelong exposure to elevated LDL-cholesterol levels, are likely to be at increased risk if exposed to PM_2.5_ particles (22). Lastly, the majority of patients presenting with an acute coronary syndrome have endothelial dysfunction, and in a 6-year follow-up study, endothelial dysfunction was an independent significant positive predictive value for major adverse cardiovascular events (hazard ratio 2.04, 95% confidence interval 1.43–2.89, *P* < 0.001) (25).

## Pharmacological prevention of cardiovascular events during wildfire smoke exposure

When exposed to wildfire toxic smoke, a likely preventive measure to decrease cardiac events is to lower PM_2.5_ particle exposure and thereby limit further endothelial dysfunction. The use of effective masks (e.g., N95 masks) during short and particularly long-lasting wildfires is one relatively inexpensive solution, but these can become uncomfortable, particularly if they need to be worn for a protracted period whilst exposed to polluting particles. Another alternative, but still an effective and practical approach, is to pharmacologically improve endothelial function to reduce the risk of cardiovascular events (26). In a recent review, Hadley and coworkers (18) stressed that an important target when preparing for the wildfire season is the prevention of cardiovascular disease. This goal can be met to some degree by the reduction of endothelial dysfunction, which can be rather rapidly achieved with pharmacological agents (27). The use of statins, which are relatively inexpensive and readily accessible, should be prioritized, as these agents acutely improve endothelial function and offer long-term stabilization of vulnerable plaques (28, 29). Several studies are showing that statin use beneficially modifies PM_2.5_ effects on inflammation and, as a result, most likely also improves endothelial function (30–35).

## Public health guidance for heart patients to prevent cardiovascular events

To assess whether pharmacological prevention is recommended to address the effects of wildfire smoke exposure, we analyzed several public guidance documents provided by the Centers for Disease Control and Prevention (CDC), USA (36), Government of Canada (37), BBC News (38), American Heart Association (AHA) press release (39), World Health Organization (WHO) Europe (40) and the Government of California (41) Table 1. The Government of Canada indicates that there is a rare risk of cardiovascular events, and that “wildfire smoke can worsen heart and lung disease”. The BBC news guidance does not address the challenges for heart patients during wildfires, while the AHA press release reminds cardiovascular patients of the risk—“People with underlying cardiovascular disease risk factors may be at risk for an acute cardiovascular event when exposed to wildfire smoke.” However, it does not mention the importance of effectively treating the underlying cardiovascular disease itself. The WHO only warns of the cardiovascular risk related to wildfire smoke exposure—“Particulate matter is capable of penetrating deep into lung passageways and entering the bloodstream, primarily resulting in cardiovascular and respiratory impacts”. Interestingly, only the CDC indirectly recommends pharmacological prevention before the wildfire season by stating, “Plan how you will protect yourself against wildfire smoke.” The CDC advises that “Before wildfire season: Talk to your healthcare provider about your heart disease”. This advice would seem to indicate that there is an unmet need to medically evaluate cardiovascular disease and to ensure that it is being effectively treated prior to wildfire smoke exposure.

**Table 1:** Public health guidance for heart patients to prevent adverse cardiovascular outcomes.

Source and title of guidance	Guidance for patients
Centers for Disease Control and Prevention (36) “*Chronic Conditions and Wildfire Smoke*”	*Before wildfire season:* Talk to your healthcare provider about your heart disease. Plan how you will protect yourself against wildfire smoke. *During a wildfire smoke event:* Think about evacuating if you have trouble breathing or other symptoms that do not get better. *After a wildfire:* Look out for any symptoms.
Government of Canada (37) ^“^*Wildfire smoke, air quality and your health”*	Less commonly, exposure to wildfire smoke can lead to stroke, heart attack and premature death.
BBC News Canada 30 June 2023 (38) “*Air quality: How to protect yourself from Canada wildfire smoke”*	No specific guidance regarding cardiovascular patients
The American Heart Association Press release 7 June 2023 (39) ^“^*Wildfires may fuel heart health hazards: smoke exposure increases cardiovascular risks”*	People with underlying cardiovascular disease risk factors may be at risk for an acute cardiovascular event when exposed to wildfire smoke. According to the American Heart Association, recognizing the signs of a heart attack or stroke are important, and if you or someone you’re with is experiencing serious symptoms, call 9–1-1 immediately.
World Health Organization (40) ^“^*Health advice: wildfires in the WHO European Region”*	Smoke from wildfires can also contribute to higher exposures to air pollution at longer distances with more long-term effects: particulate matter is capable of penetrating deep into lung passageways and entering the bloodstream, primarily resulting in cardiovascular and respiratory impacts.
Government of California (41) “*Protecting yourself from Wildfire smoke*”	Health problems related to wildfire smoke exposure can be as mild as eye and respiratory tract irritation and as serious as worsening of heart and lung disease, including asthma, and even premature death.

## Conclusion

Although not considered in current guidances, it can be concluded that pharmacological prevention using statins acutely improves endothelial function (28), and in the longer term may stabilize vulnerable plaques (29) and thereby reducing the risk of acute cardiovascular events. Only the CDC has included it in its guidance and arguably only indirectly. We encourage the various relevant authorities to place more emphasis on this neglected area to inform vulnerable cardiovascular patients exposed to wildfire smoke of their risk, to recommend that such patients seek appropriate medical care, and when medically relevant, that the use of lipid-lowering statins is considered.

Many physicians may not be necessarily aware of the harmful cardiovascular effects caused by wildfire smoke and this needs to be brought to their attention. For example, the 2021 European Society of Cardiology (ESC) Guidelines on cardiovascular disease prevention in clinical practice mentions that “Important sources of fine particles are road traffic, power plants, and industrial and residential heating using oil, coal, and wood” (42). However, wildfire smoke is not clearly mentioned in this guideline as an acute toxic exposure that can increase the risk of having a cardiovascular event. It would be beneficial in this and other respective guidance materials to inform healthcare providers and at-risk individuals, as well as the greater population, of the substantial risk that wildfire smoke poses, and that there are preventive measures that can, and should, be taken. Further prospective controlled studies with larger sample sizes are needed to confirm the association between wildfire smoke, major adverse cardiovascular events, and the role of statins in preventing them. This is particularly because there are data to show that wildfire smoke is even more toxic than many other forms of air pollution (24).
